# Generative augmentations for improved cardiac ultrasound segmentation using diffusion models

**DOI:** 10.1038/s41598-025-21938-y

**Published:** 2025-10-30

**Authors:** Gilles Van De Vyver, Aksel Try Lenz, Erik Smistad, Sindre Hellum Olaisen, Bjørnar Grenne, Espen Holte, Håvard Dalen, Lasse Løvstakken

**Affiliations:** 1 Department of Circulation and Medical Imaging, Norwegian University of Science and Technology, Trondheim, 7030 Norway; 2 SINTEF Health, Trondheim, 7034 Norway; 3 St. Olavs hospital, Trondheim, 7030 Norway

**Keywords:** Cardiac segmentation, Ultrasound, Generative AI, Diffusion models, Echocardiography, Computer science, Information technology, Software

## Abstract

One of the main challenges in current research on segmentation in cardiac ultrasound is the lack of large and varied labeled datasets and the differences in annotation conventions between datasets. This makes it difficult to design robust segmentation models that generalize well to external datasets. This work utilizes diffusion models to create generative augmentations that can significantly improve diversity of the dataset and thus the generalisability of segmentation models without the need for more annotated data. The generative augmentations are applied in addition to regular augmentations. A visual test survey showed that experts cannot clearly distinguish between real and fully generated images. Using the proposed generative augmentations, segmentation robustness was increased when training on an internal dataset and testing on an external dataset with an improvement of over 20 millimeters in Hausdorff distance. Additionally, the limits of agreement for automatic ejection fraction (EF) estimation improved by up to 20% of absolute EF value on out of distribution cases. These improvements come exclusively from the increased variation of the training data using the generative augmentations, without modifying the underlying machine learning model.

## Introduction

Ischaemic heart disease is the leading cause of death worldwide, accounting for 13% of all global fatalities and is the fastest rising cause of death since the beginning of the century^1^. Ultrasound imaging, being cost-effective, safe, and real-time, is the most common technology used for evaluating heart function. Accurately delineating the LV and myocardium (MYO) directly enables the extraction of clinical measurements of heart function, such as EF and global longitudinal strain (GLS). However, measurements are labour intensive and even for experienced cardiologists there is high operator-related variability, with a coefficient of variation between 6 and 11%^2^. Automating the process of extracting clinical measures from the recordings reduces inter-observer variability and allows measurements over multiple heart cycles, as recommended in the guidelines^3–6^, without additional labour.

Due to data protection regulations and the labour-intensive process of data curation and annotation, only a limited number of open datasets are available, and those that exist are of limited size^7–9^. Although guidelines for tracing cardiac structures such as the endocardium exist^3^, the amount of noise and artifacts in ultrasound, as well as structures such as the trabeculae, make tracing of the true endocardial wall challenging. Thus, if one would ask multiple experts to annotate the endocardial wall, it would result in multiple different annotations. This variability in annotation preference and inter-vendor differences means that combining datasets from different centres should be done with care. This is in stark contrast with the field of computer vision for natural images, where large, labelled datasets are common. The sparsity of annotated data with a consistent annotation protocol creates unique challenges for deep learning networks, particularly in terms of robustness and generalization^10^.

Several works have explored using generative AI to improve segmentation in cardiac ultrasound. the idea is to generate images conditioned on segmentation masks to create image-label pairs for training segmentation models. Gilbert et al.^11^ and Tiago et al.^12^ use generative adversarial networks (GANs) for image generation from anatomical models in 2D and 3D respectively. Stojanovski et al.^13^ developed a conditional Denoising Diffusion Probabilistic Models (DDPMs) that generates images from segmentation masks. They apply basic transformations to the segmentation masks of an existing labelled dataset and then feed these to the conditional DDPM to generate new images. Jafari et al.^14^ and Tiago et al.^15^ address domain translation, using CycleGANs and adversarial diffusion models respectively.

Generative models trained with conditioning on a segmentation mask have the inherent limitation that they can only be trained on labelled data. In cardiac ultrasound, typically only a portion of the data is labelled with pixel-wise segmentation masks as the annotation process is labour intensive. Another limitation of current works using conditioning is that the generated images have no guarantee to correspond exactly to the labelled mask, potentially lowering the quality of the image-label pair. This is especially important for ultrasound cardiac segmentation. Pixel accurate delineation of the MYO can be non-trivial, and experts in the field are needed to create high-quality annotations. This guarantee of high-quality labels is lost when original pixels of the MYO border are generated. This motivates our approach to generate only the pixels surrounding the structure of interest.

DDPMs are a type of generative model that learns to approximate a data distribution by reversing a gradual, multi-step noise addition process. They were first introduced by Sohl-Dickstein et al.^16^, and subsequently improved upon by Ho et al.^17^, and Nichol and Dhariwal^18^. The latter showed that diffusion models can outperform GAN based models for image synthesis^19^. Diffusion models are also appealing as they do not suffer from the training difficulties often encountered with GANs^20^.

In the forward process during training, denoising diffusion probabilistic models (DDPMs) define a diffusion process that transforms an initial image $x_0$ into approximately white Gaussian noise $x_T \sim \mathscr {N}(0, I)$ over *T* timesteps. Each forward step in this process is defined by

$$q(x_t \mid x_{t-1}) = \mathscr {N}\left( x_t; \sqrt{1 - \beta _t} x_{t-1}, \beta _t I \right)$$

In this equation, noise is added to $x_{t-1}$ using Gaussian noise with variance $\beta _t$ and the previous latent $x_{t-1}$ is scaled by $\sqrt{1 - \beta _t}$ according to a variance schedule^17^.

The learning goal in a DDPM is to approximate the the reverse process $q(x_{t-1} \mid x_t, x_0)$, which is another Gaussian of which the mean and variance that can be written in closed form^17^. This reverse process is modeled by

$$p_\theta (x_{t-1} \mid x_t) = \mathscr {N}\left( x_{t-1}; \mu _\theta (x_t, t), \Sigma _\theta (x_t, t)\right)$$

of which the mean $\mu _\theta (x_t, t)$ and optionally the variance $\Sigma _\theta (x_t, t)$ are estimated by a neural network, typically a U-Net^21^. The mean and variance can then be learned by optimizing the variational bound on the negative log likelihood, which boils down to minimizing the Kullback-Leibler (KL) divergence between the two Guassian distributions $q(x_{t-1} \mid x_{t},x_{0})$ and $p_\theta (x_{t-1} \mid x_t)$. Because the mean and variance of the target distribution have closed expressions, the KL divergence can be computed analytically using the predicted mean and variance, allowing for direct optimization via gradient descent^17^.

The learning methodology of the DDPM has been progressively refined. The DDPM of Ho et al. estimates the noise of the Gaussian target distributions and uses it to calculate the latent of the previous step in the reverse process of the diffusion model. The variance is kept constant with a hyperparameter^17^. Nichol and Dhariwal introduced several improvements to the method of Ho et al. These most important improvements are estimating the variance in addition to the mean, improving the noise scheduler, and using importance sampling during training^18^.

RePaint^22^ is a method that use a DDPM to replace specific regions of an image, as controlled by a given mask. During inference, the method takes as input an image and a mask. After each step of the reverse diffusion process, the part of the generated image that falls outside of the mask is replaced by the corresponding parts of the input image, with appropriate noise added for that step in the diffusion process. This ensures that only the image regions inside the mask are synthesized, while those outside are left unchanged, and thus allows the unconditionally trained DDPM to be guided by the input image during inference.

Several studies have investigated generative models for segmentation, but state-of-the-art methods in cardiac ultrasound, and also medical imaging, have not widely adopted them. Instead, leading approaches to cardiac ultrasound segmentation continue to rely on U-Net variants^21,23–26^. We also use a U-Net–based model for segmentation in this work.

In this work, we develop a method that augments a labelled cardiac segmentation dataset using an unconditional diffusion model. Our method has two unique advantages. First, since it uses an unconditionally trained DDPM, a dataset can be augmented using a generative model trained on an unlabelled dataset or a dataset with different annotation conventions. Second, since our model does only alter the surroundings of the segmentation masks, the most crucial parts of the image remain untouched. Thus, fine annotation subtleties and details in the original image-label pair are not affected by the generative model. This distinguishes our approach from the work of Kupyn and Rupprecht^27^ who repaint the semantic object itself.

The proposed method is most effective for improving the performance of a segmentation model trained on a dataset with limited variation in terms of acquisition and positioning of the LV in the image. More specifically, this work uses the HUNT4Echo^28^ dataset which has annotations of high quality which are time-consuming to obtain. The recordings in this dataset are LV-focused: the recordings were obtained following the clinical guidelines and maximize the area of the ventricle in the image^3^. Thus, the dataset has limited variety in terms depth, sector width, and positioning of the ventricle in the image. This leads to poor performance of segmentation models trained on this dataset when tested on an external dataset with more variation like the public CAMUS^7^ dataset. In this work, we explore whether generative augmentations can be used to enrich this limited but high-quality dataset so that the resulting models can generalize better to datasets with more variation.

The contributions of this paper are:

- A method for creating realistic generative augmentations of cardiac ultrasound images using DDPMs.
- A blinded expert evaluation of the realism of fully generated images.
- An ablation study that evaluates the effect of the proposed generative augmentations on the segmentation accuracy.
- A clinical evaluation of the effect of generative augmentations on automatic segmentation-based EF measurements.

## Materials and methods

### Datasets

The **HUNT4Echo dataset **^28^, part of the Helse Undersøkelsen i Nord-Trøndelag study, is an ultrasound dataset of 2,211 volunteers of LV-focused apical 2-chamber (A2C) and apical 4-chamber (A4C) views, acquired using a GE Vivid E95 scanner. Each recording includes three cardiac cycles. The dataset is an in-house dataset from St. Olavs hospital in Trondheim, Norway and is not publicly available. All participants signed informed consent for participation and the use of data in research^29^. The regional ethics board (REB) approved all parts of the study (REC ID 2018/2416). The datasets used and analysed during the current study is not public available. Håvard Dalen (havard.dalen@ntnu.no) is the contact point for the HUNT4Echo data. All methods were carried out in accordance with relevant guidelines and regulations. The **model development set** is a subset that includes single-frame segmentation annotations for both end-diastole (ED) and end-systole (ES), providing pixel-level labels for the LV, left atrium (LA), and MYO. The model development set consists of 1058 annotated ED and ES frames of 529 recordings from 311 patients. The **EF set** is a subset disjunct with the model development set of 1900 patients with reference biplane LV volumes in ED and ES. The volumes were obtained following current clinical guidelines by manual tracing and using Simpson’s method of discs in the clinically approved EchoPAC software from GE HealthCare on the HUNT4 recordings. We use EchoPac v203 https://services.gehealthcare.com/gehcstorefront/p/EZ100730-19.

The **CAMUS dataset**^7^ is a publicly available dataset containing single cycle recordings from 500 patients, acquired using a GE Vivid E95 scanner (GE Vingmed Ultrasound AS, Norway). The dataset contains one A2C and one A4C recording for each patient and annotations for both the ED and ES frame in each recording, resulting in a total of 2000 image-annotation pairs. Like the HUNT4 dataset, the annotations are pixel-level LV, LA, MYO. The training, validation and test set contain 400, 50 and 50 patients respectively.

**Fig. 1:**
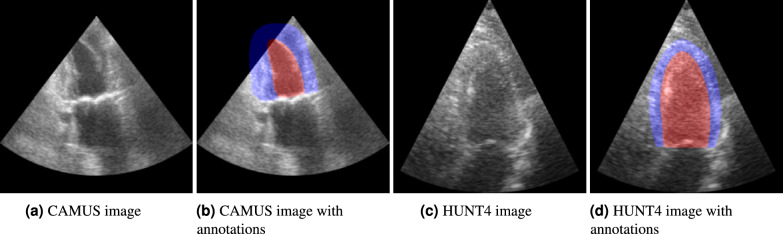
Example frames from the CAMUS and HUNT4 datasets. The CAMUS and HUNT4 dataset contain the same cardiac views, but the frames in HUNT4 are consistently LV-focused, while those in CAMUS are not. The annotations conventions are also different in both datasets, which can be seen clearly in the thickness of the annotated MYO (blue).

The HUNT4 model development set and the CAMUS dataset should be combined with care due to differences in annotation conventions. For example, the MYO is consistently annotated as significantly thicker in CAMUS compared to HUNT4. Fig. 1 illustrates this. The segmentation models in this work only segment the LV and MYO labels and the experiments only evaluate on the LV. We elaborate on this choice in the Discussion.

**Fig. 2:**
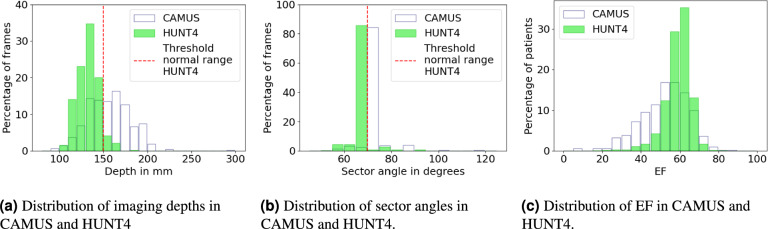
Characteristics of the CAMUS and HUNT4 datasets.

**Fig 3:**
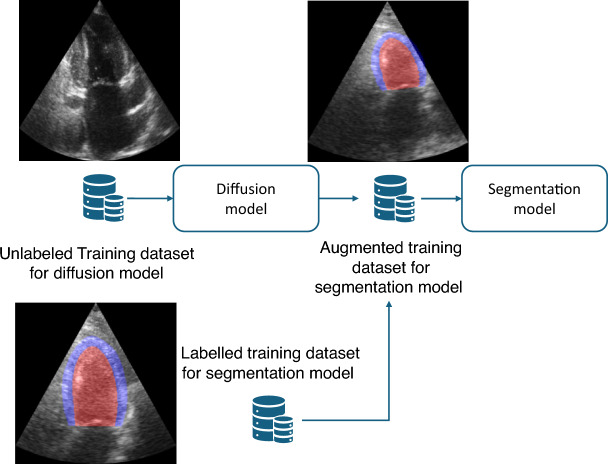
Flow chart of the proposed method. The training dataset for the diffusion model does not have to be labelled. If the training dataset for the diffusion model contains more variety than the labelled training dataset for the segmentation model, generative augmentations produce training samples with more diversity.

The recordings in the HUNT4 study follow the clinical guidelines for quantitative measurements and maximize the area of LV in the image by adjusting the depth^3^. This is not the case for CAMUS, resulting in less standardized images. The top part of Fig. 10 shows the heatmaps of pixels labelled as LV in both datasets, illustrating that the LV occupies a larger portion of the image in HUNT4 compared to CAMUS. This is a consequence of the distribution of acquisition depth and width, shown in Fig. 2a and b. The CAMUS dataset also has a larger variety in terms of EF, as shown in Fig. 2c. Table 1 shows the most important characteristics of the CAMUS and HUNT4 datasets. This work uses generative augmentations to enrich the HUNT4 dataset so that the resulting models can generalize better to other datasets, such as CAMUS. Figure 3 shows a flow chart of the proposed method.

**Table 1:** Characteristics of the CAMUS and HUNT4 datasets. For HUNT4, the charasetristics of the model development set and EF set are identical. The numbers shown are mean and standard deviation.

Dataset	Depth	Sector angle	EF	BMI	Participants
CAMUS	$151.6 \pm 24.5$ mm	$75.2 \pm 5.8^\circ$	$52.5 \pm 12.3 \%$	N/A	Healthy and unhealthy patients
HUNT4	$127.8 \pm 12.2$ mm	$65.5 \pm 4.0^\circ$	$59.1 \pm 6.6\%$	$26.5 \pm 4.0$	Mostly healthy volunteers

Our study does not include segmentation of the LA because it is often not fully visible in the original scan. This is especially true for the LV-focused HUNT4 images. When using generative augmentation, particularly depth augmentation, the diffusion model can generate parts of the LA that were missing in the original scan. This creates problems because the original labels only correspond to the visible parts of the LA in the original image. Therefore, we restrict ourselves to the LV and MYO in this work.

### Training of the diffusion model

This work uses the DDPM described by Nichol and Dhariwal^19^. It consists of a 44 million parameter U-Net with 4000 diffusion steps. The supplementary material contains more details on the network and training setup.

**Fig 4:**
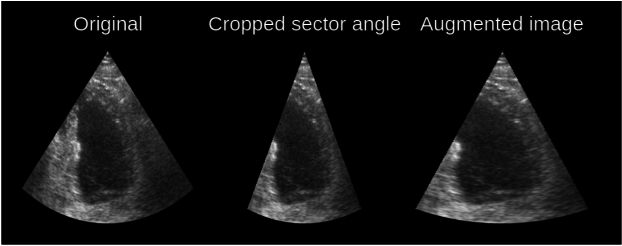
Sector width preprocessing regular augmentation on CAMUS performed before training of the DDPM. This was done to handle the lack of sector width variation in the CAMUS dataset. The sector angle is first reduced and then the sector is stretched back to 256 by 256 pixels. This step increases the variation in sector widths in the RePaint training set. These regular augmentations are applied solely during the training of the diffusion model and are not part of the generative augmentations described below.

The goal of the proposed method is to enrich the variety of annotated datasets, so the training dataset of the DDPM should be diverse enough. The CAMUS dataset is mostly diverse enough for this purpose. However, since the majority of sector angles in the acquisitions is around $75^\circ$, a DDPM trained on the CAMUS dataset struggles to generate images with a different sector angle. Therefore, we apply a preprocessing regular augmentation step that randomly narrows the sector angle by up to 20 degrees, removing pixels along the peripheral scan lines, and then stretches the cut sector back to $256\times 256$ pixels, as illustrated in Fig. 4. Additionally, to preserve data variety while reducing the training dataset size, only every Nth frame is used, where N is a random number between 8 to 12 frames. This reduces the training set size while preserving the variation since consecutive frames are often very similar due to the high acquisition frame rate.

The generative model in this work is the RePaint model as described by Lugmayr et al^22^, where the training and sampling process of the DDPM is replaced by the improved DDPM as described by Nichol and Dhariwal^18^.

### Generative augmentations

**Fig 5:**
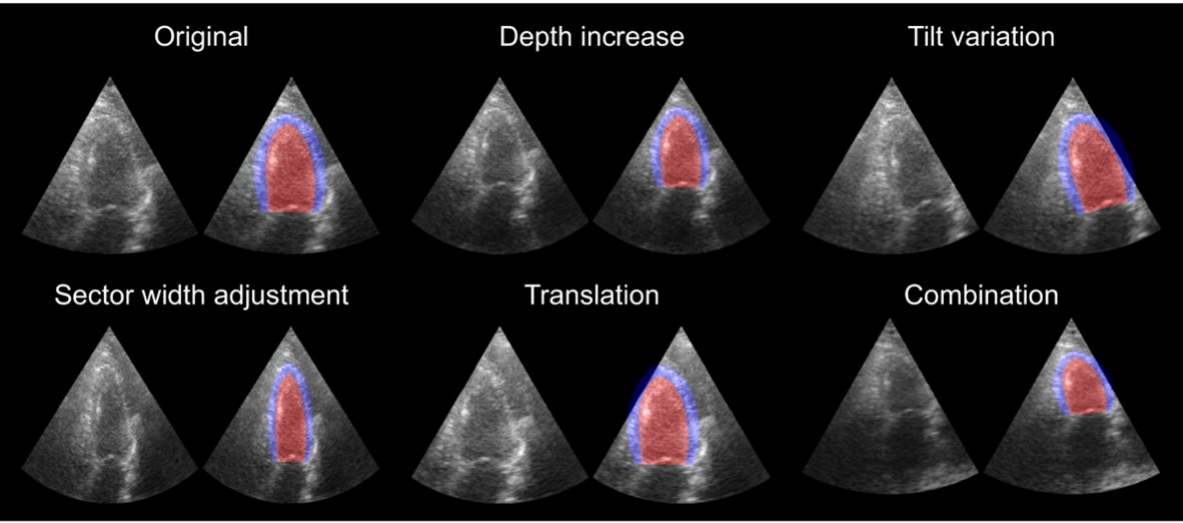
Examples of the generative augmentations types used in this work. All the examples are generated from the same original image shown in the top-left corner. The MYO is indicated in blue. The LV is indicated in red.

**Fig 6:**
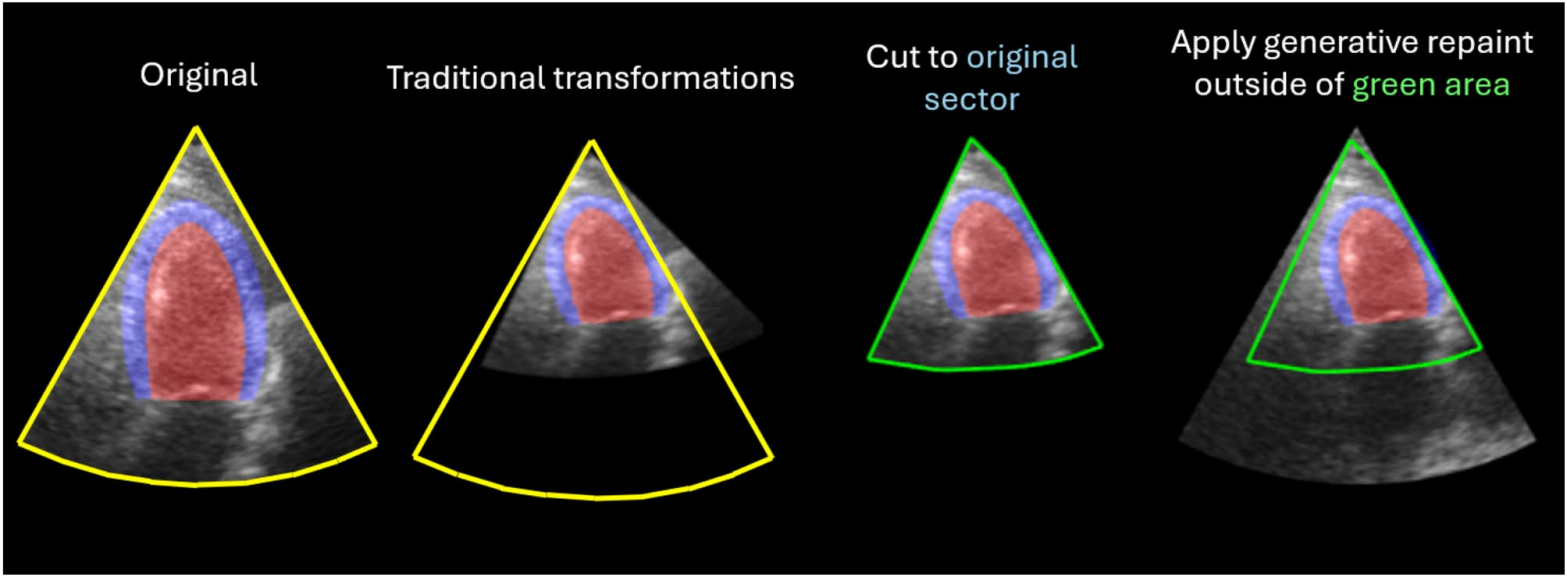
Process of creating generative augmentations. First, the frame is transformed with the transformation described in the ’Generative augmentations’ subsection. Then, the pixels outside the original sector are turned black. Finally, the generative model repaints all black pixels re-creating a complete sector in the process. The yellow contour delineates the original sector. The green contour delineates the part that is kept from the original image. The original sample is taken from the HUNT4 development set. The masks of the LV lumen and MYO are painted in red and blue respectively.

To apply generative augmentations to a cardiac ultrasound image, the image is first transformed using random depth, tilt, width and translation transformations as described below and illustrated in Fig. 5. Then the trained diffusion model is applied to synthesize pixels outside of original input image using the RePaint method. Fig. 6 shows this process. Each image is transformed and augmented five times. The augmented training dataset then contains the original image and the five augmented samples.

- **Depth increase**: the depth of the original image is increases randomly by $\lambda =[0,150]$ pixels by adding black pixels at the bottom of the image and then resizing to $256\times 256$.
- **Tilt variation**: the original image is rotated with a random angle around the sector tip by $\theta$ degrees, with $-30^{\circ }<\theta <30^{\circ }$.
- **Sector width adjustment**: the width of the original image is multiplied by a factor $\lambda$. If $\lambda>0$, the image gets stretched out horizontally and cropped back to $256\times 256$. If $\lambda <0$, the image gets squeezed into the center. Here, $0.5<\lambda <1.5$. This generative augmentation is similar to the work of Gazda et al.^30^. Resizing to $256\times 256$ distorts the image.
- **Translation**: the original image is shifted by a vector with a random angle and length $\lambda$, with $0<\lambda <50$ pixels. The result is cropped back to $256\times 256$ pixels.
- **Combination**: all of the above generative augmentations are applied with a 50% chance.

### Survey

To evaluate the realism of the generated images, a survey was conducted with three groups of human evaluators. The first consisted of three senior cardiologists certified by the EACVI in transthoracic echocardiography, each with over 15 years of experience and more than 10,000 examinations. The second group included four clinical researchers. The last group consisted of three engineers specializing in cardiac ultrasound. Each participant was asked to distinguish real from synthetic images. The real images were sampled randomly from the CAMUS dataset. The synthetic images were generated by the DDPM trained on the CAMUS dataset. The participants were given 50 pairs of images and were told one of the images in each pair was synthetic. The participants than had to select the synthetic image and give an explanation for their selection. Additionally, 5 of the 50 pairs contained two real images without the knowledge of the participants.

### Segmentation ablation study

The goal of the ablation study is to evaluate how different types of generative augmentations, described in the ‘Generative augmentations’ subsection, improve segmentation performance. We use the nnU-Net framework^24,31^ as the segmentation model, applying its default configuration but skipping cross-validation. Instead, a single model is trained on the dataset splits defined in the ‘Datasets’ subsection. We do not use cross-validation for training. Instead, the official training, validation, and test splits of the CAMUS dataset were used. This makes our results comparable to other studies using the CAMUS dataset. The supplementary material contains more details on the segmentation model architecture and training setup.

For each type of generative augmentation, we create a training and validation set that combines all the original images frames with five randomly augmented images generated from each original image. Additionally, we use a baseline augmentation that applies the same transformations as the combination generative augmentation but does not repaint the missing parts, leaving those areas black. This allows us to evaluate whether the repainting actually compared to just applying the transformation augmentations. The resulting sets are then used to train separate models using the nnU-Net framework. The nnU-Net framework applies its regular annotations listed in the supplementary material on top of the generative augmentations described here. We evaluate each model’s performance on the original test sets of both CAMUS and HUNT4 datasets.

### Clinical evaluation on HUNT4

This experiment compares the automatic segmentation-based EF trained with different augmented datasets to the manually measured EF using the clinical EchoPAC software from GE HealthCare. The automated estimation of EF is the same procedure as in previous works^26,32^ and follows the steps outlined by the clinical guidelines for manual EF estimation^3^:

1. Use the timing network proposed by Fiorito et al.^33^ to detect the ED and ES frames of each cardiac cycle for both the A2C and A4C recordings of the same patient. Thus, the view is manually labelled during acquisition, while the timing is obtained through deep learning.
2. Use the segmentation network to segment all ED and ES frames.
3. Use the modified Simpson method to calculate the LV volume in ED and ES using the A2C and A4C frames. Each A2C frame is combined with each A4C frame for each cardiac cycle and the results are averaged.
4. Calculate $$EF=\frac{{ED\,volume}-ES\,volume}{ED\,volume}$$

When the segmentation fails for all heart cycles, the algorithm can not extract an EF value and the exam is omitted from analysis, similar to our previous work^26^. The excluded images had unsuitable low image quality or were out of distribution, usually due to having an incorrect cardiac view^26^.. For a fair comparison between the models trained with and without generative augmentations, we only include exams for which both versions manage to extract an EF value. Our results include 1872 out of 1900 patients in the HUNT4 EF set, which corresponds to a feasibility of 98.5%.

### Real-time demo

**Fig. 7:**
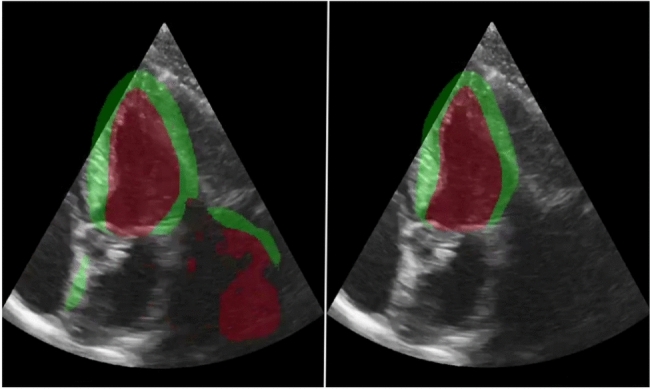
Screenshot of the real-time demo application. The left side shows the segmentation of the segmentation model trained in the usual way. The right side shows the segmentation of the same model trained with the combination of all generative augmentations. The figure shows a screen capture of the demo, recorded live. Therefore, there is no reference available.

To visually demonstrate the differences between the model trained with and without generative augmentations with varying acquisition parameters, a real-time application was created using the FAST framework^34^. The application shows the segmentation output of the segmentation model trained without generative augmentations and the model trained with the combination of all generative augmentations side by side in real-time while streaming from a GE HealthCare Vivid E95 scanner. Fig. 7 shows a screenshot of the application. The video is available at https://doi.org/10.6084/m9.figshare.28219919. These clearly demonstrate an increased segmentation model robustness in terms of acquisition parameters such as depth, angle and LV positioning.

### Code availability

The generative augmentation tool is available as an open-source Python library with pre-trained models on CAMUS at https://doi.org/10.5281/zenodo.17009461

## Results

### Evaluation of generated images

**Fig. 8:**
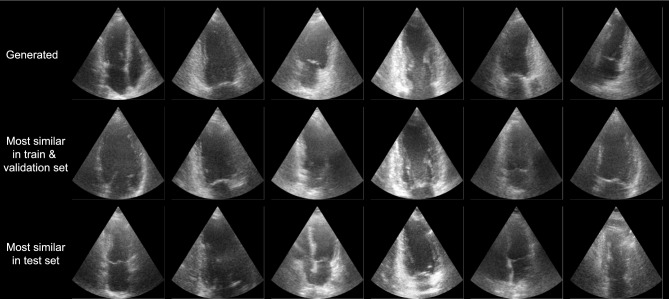
Generated samples, together with most similar cases in the train and validation set and the test set of the CAMUS dataset, based on SSIM^40^.

The ImageNet Fréchet inception distance (FID)^35^ and inception score (IS)^36^ of the diffusion model are 23.87 and 1.47 respectively. However, these metrics can give misleading results for generative models that are not trained on ImageNet^37–39^. To qualitatively assess the performance of the model, Fig. 8 shows random samples generated together with the most similar cases from the CAMUS dataset identified automatically using the structural similarity index measure (SSIM)^40^. The generated image shows different anatomy and a different cardiac phase than the real images in the train and test set, while still looking realistic. This shows the model does not simply memorize cases from the training set, and produces realistic and varied samples.

### Survey results

**Fig. 9:**
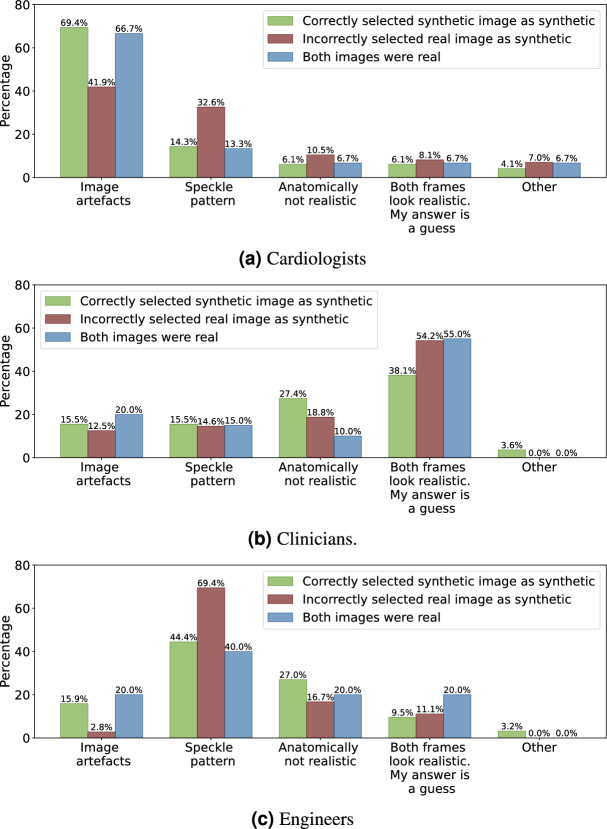
Explanations given during the survey by the different expert groups.

On the 45 pairs with one real and one synthetic image, participants correctly identified the synthetic image 56.4% of the time. When broken down by group, cardiologists achieved an accuracy of 63.7%, while clinical researchers and engineers both identified the correct frame 53.3% of the time. Figure 9 shows the explanations given when the participants correctly identified the synthetic frame, when they were wrong, and when both frames were real in the 5 cases mentioned above.

Using a binomial test with a significance level of 5%, the accuracy of the cardiologists was found to be statistically significantly higher than random guessing ($P=0.09\%$). However, the engineers and clinical researchers in the survey did not show statistically significant higher accuracy compared to random guessing ($P=24.6\%$). Table 2 lists Average Cohen’s Kappa agreement^41^ between each group.

**Table 2:** Average Cohen’s Kappa score^41^ between different subgroups during the survey.

	Cardiologists	Clinicians	Engineers
Cardiologists	0.355	-	-
Clinicians	0.039	0.083	-
Engineers	0.182	0.069	−0.022

### Results of segmentation ablation study

**Fig. 10:**
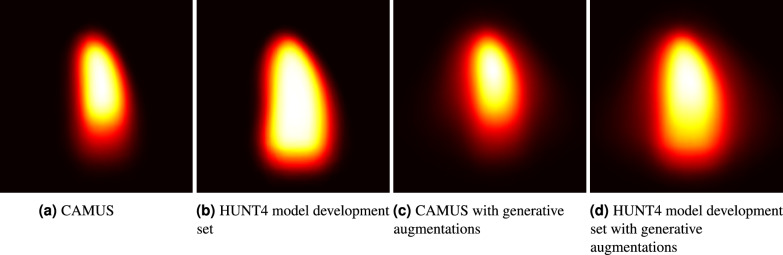
Heatmaps of pixels belonging to the LV after resizing to $256\times 256$. This illustrates the difference in scan depth variation and LV positioning in the two datasets.

Table 3 shows the results of the ablation study on the CAMUS dataset, using Dice score and Hausdorff distance as metrics. Figure 10 shows the heatmaps of pixels belonging to the LV after applying the combination of all generative augmentations. Comparing these to the original illustrates that the generative augmentations increase the variety of LV location in the image.

The increase in segmentation accuracy of the HUNT4 model on CAMUS originate mostly from an improvement in segmentation accuracy for samples outside the HUNT4 image distribution. Table 4 lists the segmentation results for the HUNT4 models on different subsets of CAMUS. The subsets are based on depth and sector angle cut-off values visualized in Fig. 2a and b.

**Table 3:** Segmentation results of the ablation study using different datasets (HUNT4 and CAMUS) for training and testing. For all experiments, regular augmentations are applied in addition to the generative augmentations (see supplementary material).The Dice score and Hausdorff distance are only for the LV lumen label. We elaborate on this choice in the Discussion. Since the two datasets have been annotated by different experts with different annotation conventions, there is a considerably lower segmentation accuracy when the training and test sets are different. $^*$The last column tests whether the differences in Dice and Hausdorff scores respectively are statistically significant (p < 0.05) compared to the version without generative augmentations using the Wilcoxon signed-rank test^42^. The first yes or no applies to the Dice score, the second to the Hausdorff distance. Italic or underline indicate positive or negative significance respectively. Since the ablation study tests six different augmentations settings, Bonferroni correction with $m=6$ is applied.

Training set	Test set	Generative Augmentations	Dice score	Hausdorff distance (mm)	Statistically significant$^*$
HUNT4	CAMUS	None	0.802 ± 0.15	29.03 ± 26.01	N/A
		Depth increase	0.887 ± 0.05	**7.49 ± 3.25**	*yes, yes*
		Tilt variation	0.829 ± 0.14	17.31 ± 20.98	*yes*, no
		Sector width	0.847 ± 0.11	21.36 ± 23.84	*yes*, no
		Translation	0.840 ± 0.12	16.55 ± 19.71	*yes, yes*
		Combination	**0.887 ± 0.05**	8.17 ± 5.32	*yes, yes*
		Combination without repaint	0.810 ± 0.15	26.90 ± 25.07	*yes*, no
CAMUS	CAMUS	None	0.943 ± 0.03	4.46 ± 2.52	N/A
		Depth increase	0.945 ± 0.03	**4.27 ± 2.34**	no, no
		Tilt variation	0.945 ± 0.03	4.30 ± 2.43	no, no
		Sector width variation	**0.946 ± 0.03**	4.34 ± 2.41	no, no
		Translation	0.944 ± 0.03	4.44 ± 2.43	no, no
		Combination	0.944 ± 0.03	4.37 ± 2.43	no, no
		Combination without repaint	0.934 ± 0.03	5.39 ± 2.85	yes, yes
Gilbert et al.^11^	CAMUS	N/A	0.812± 0.054	N/A	N/A
Stojanovski et al.^13^		N/A	0.886± 0.058	N/A	N/A
HUNT4	HUNT4	None	0.952 ± 0.02	3.34 ± 1.21	N/A
		Depth increase	0.954 ± 0.02	3.24 ± 0.99	no, no
		Tilt variation	0.954 ± 0.02	3.38 ± 1.06	no, no
		Sector width variation	0.953 ± 0.02	**3.23 ± 1.00**	no, no
		Translation	0.954 ± 0.02	3.32 ± 0.97	no, no
		Combination	**0.954 ± 0.02**	3.31 ± 0.99	no, no
		Combination without repaint	0.947 ± 0.02	4.14 ± 1.85	yes, yes
CAMUS	HUNT4	None	0.886 ± 0.04	6.70 ± 1.81	N/A
		Depth increase	0.891 ± 0.04	6.55 ± 1.84	*yes*, no
		Tilt variation	0.887 ± 0.04	6.69 ± 1.91	no, no
		Sector width variation	0.892 ± 0.04	**6.54 ± 1.78**	*yes*, no
		Translation	0.890 ± 0.04	6.55 ± 1.83	*yes*, no
		Combination	**0.892 ± 0.04**	6.59 ± 1.82	*yes*, no
		Combination without repaint	0.875 ± 0.04	7.71 ± 2.11	yes, no

**Table 4:** Segmentation results on different CAMUS subsets for a segmentation model trained on HUNT4 without generative augmentations and with the combination of all generative augmentations. $^*$The last column tests whether the difference in Dice and Hausdorff scores respectively are statistically significant (p < 0.05) compared to the version without generative augmentations using the Wilcoxon signed-rank test^42^. The first yes or no applies to the Dice score, the second to the Hausdorff distance. Italic or underline indicate positive or negative significance respectively.

Training dataset	CAMUS Test subset	Dice score	Hausdorff distance (mm)	Statistically significant$^*$
HUNT4 without generative augmentations	Depth $< 150$ mm ($n=1088$)	0.855 ± 0.11	14.48 ± 16.61	N/A
	Depth $\ge 150$ mm ($n=912$)	0.729 ± 0.18	45.83 ± 30.19	N/A
	Sector angle $< 70^\circ$ ($n=146$)	0.869 ± 0.10	12.47 ± 16.47	N/A
	Sector angle $\ge 70^\circ$ ($n=1854$)	0.792 ± 0.16	30.06 ± 28.80	N/A
HUNT4 with generative augmentations	Depth $< 150$ mm ($n=1088$)	**0.893 ± 0.05**	**7.45 ± 3.80**	*yes, yes*
	Depth $\ge 150$ mm ($n=912$)	**0.886 ± 0.07**	**9.34 ± 8.37**	*yes, yes*
	Sector angle $< 70^\circ$ ($n=146$)	**0.893 ± 0.05**	**7.11 ± 3.10**	*yes, yes*
	Sector angle $\ge 70^\circ$ ($n=1854$)	**0.890 ± 0.07**	**8.40 ± 6.56**	*yes, yes*

### Results of clinical evaluation on HUNT4

Similar to the segmentation results, the performance gains of the HUNT4 model originate mostly from an improvement in segmentation accuracy for frames outside the normal range. Figure 13 shows the Bland-Altman plots comparing the manual reference EF with the automatic EF for segmentation models trained with and without generative augmentations for data both inside and outside of the HUNT4 acquisition normal range of depth $> 150$mm and sector angle $> 70^\circ$. The supplementary material contains additional analysis of automatic EF and also evaluates automatic on CAMUS.

## Discussion

### Survey analysis and hallucinations

The results of the survey shows that the DDPM can generate highly realistic ultrasound images that are hard to distinguish, even for ultrasound experts. Although senior cardiologists could distinguish synthetic images better than random guessing, they were still correct only in 63.7% of the cases. The analysis of agreement between the subgroups confirms that the cardiologists have a moderate agreement between each other, while the agreement for the other groups is low.

**Fig. 11:**
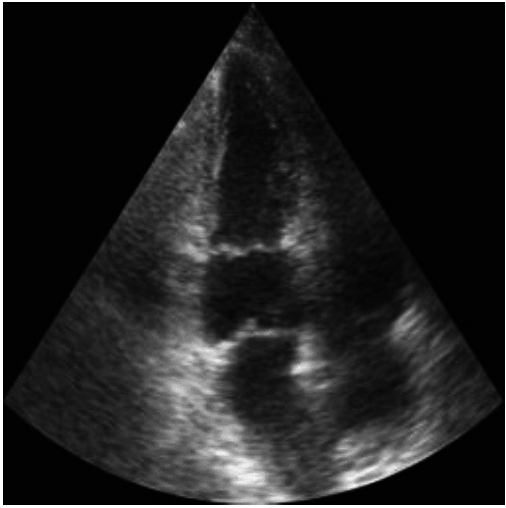
A problematic hallucination by the diffusion model, generating a second mitral valve below the true mitral valve.

Generative AI can create unrealistic and anatomically incorrect images, also known as hallucinations, and the DDPM in this work is no exception. Figure 11 shows an example of a problematic hallucination in which the model creates an additional mitral valve. In this case, the generative augmentation would add noise to the training data. How frequently these kinds of hallucinations happen depend heavily on the input given as starting point to the model. When the model generates a full image from scratch as in the survey, it does not generate obvious hallucinations. However, obvious hallucinations become more and more frequent as the model starts from more heavily transformed images that are not represented in the training data. This is especially the case when the input images are highly unrealistic.

For segmentation augmentations, the reference mask regions are most critical, while the background is less important. Although imperfectly generated surroundings are usually acceptable, realism still matters. Table 3 shows that in three of four dataset combinations, baseline augmentations with black backgrounds actually reduce performance, possibly because they make images less realistic without adding useful information. On the other hand, realistic, generated surroundings improve results compared to the baseline augmentations.

### Differences in reference annotations

While the segmentation models predict both LV and MYO, the experiments only evaluate on the LV. The experiments do not evaluate on the MYO because there is a notable difference between the annotation conventions between HUNT4 and CAMUS. The annotations in the CAMUS dataset consistently label the MYO notably thicker than the HUNT4. Figure 1 shows an example of this. There are also differences in annotation conventions for the LV lumen, but these are less pronounced than for the MYO. Nevertheless, the Dice score is on average.08 lower when training on CAMUS and testing on HUNT4 and vice versa, as shown in Table 3.

### Analysis of ablation study and clinical evaluation

**Fig. 12:**
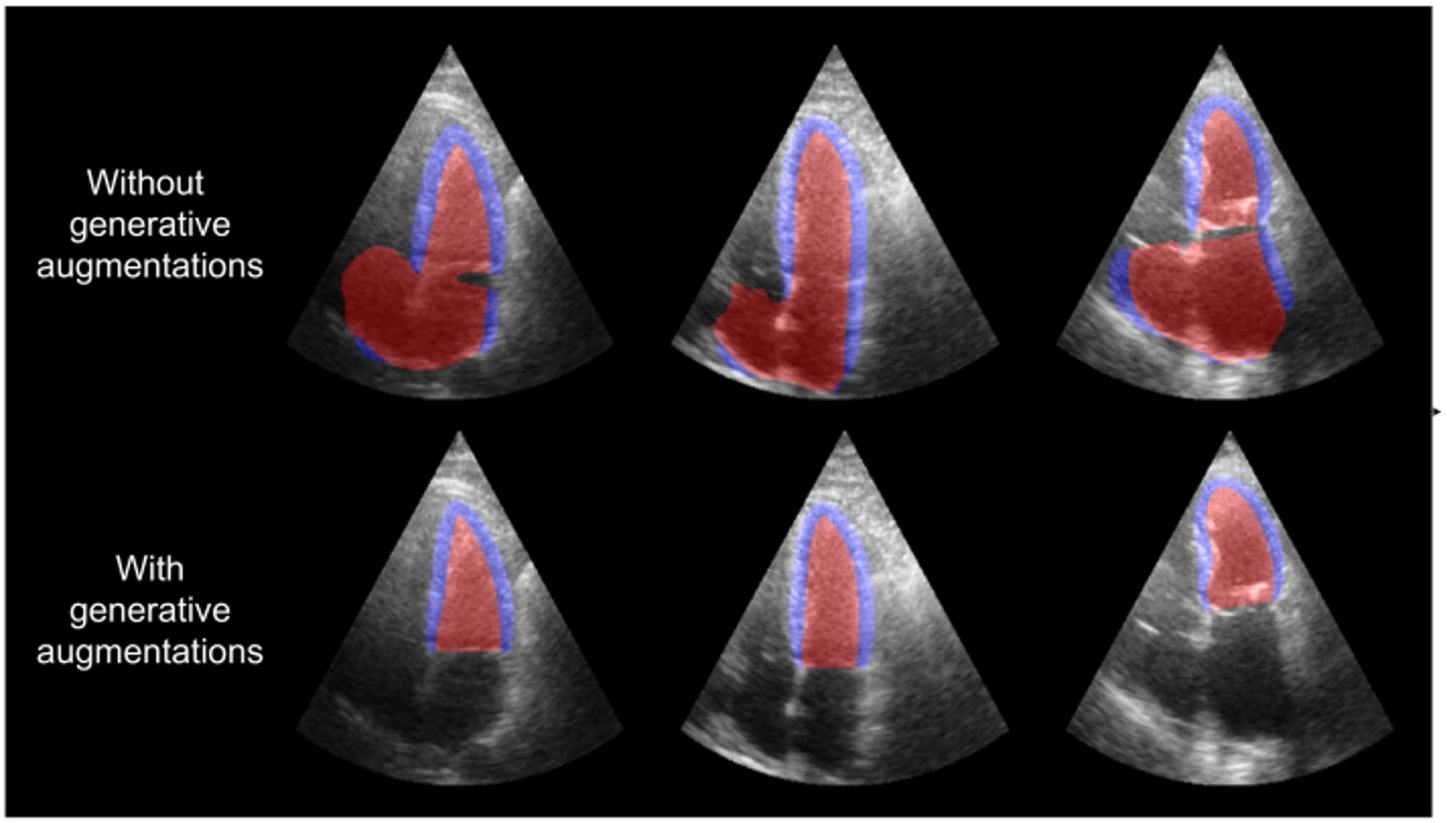
Segmentation results for HUNT4 study participants with the largest difference in automatic EF for models with and without generative augmentations. The model trained without generative augmentations fails to correctly segment the LV for frames with increased depth due to the lack of such images in the training set.

The clinical evaluation on HUNT4 showed the generative augmentations lead to narrower limits of agreement with the reference in terms of EF. The reduction in limits of agreement originates from a reduction in segmentation failures (outliers). Figure 12 shows visualizations of segmentation outputs for HUNT4 study participants where the segmentation models with and without generative augmentations lead to the largest differences in automatic EF. In the training set of the HUNT4 development set, all views are LV-focused meaning that a shorter scan depth is used so that the LV covers most of the scan sector. LV-focused views are used because this aligns with clinical guidelines, which recommend optimizing the view to ensure the LV is clearly and fully visualized for accurate assessment. However, in practice it can be hard to get a standardized view. Without the generative augmentations, the model overfits on LV-focused views and thus often fails to segment the LV correctly when views are not focused on the LV. This explains why the generative depth augmentation are the most successful generative augmentation in the ablation study.

The clinical evaluation shows that the bias changes depending on which dataset the segmentation model was trained on. This bias can be corrected for by measuring its magnitude on a subset of the target domain and adjusting accordingly on new, unseen data. The reason for the change of bias can be the data distribution in the training set, the annotation conventions, or the methodology of the tools used for manual measurement^4^. An in-depth analysis of the bias is out of scope for this work.

Both the ablation study and the clinical evaluation on HUNT4 show that the model trained on HUNT4 benefits the most from the generative augmentations. The Model trained on HUNT4 and tested on CAMUS improved by.08 when adding generative augmentations, while the model trained on CAMUS and tested on HUNT4 improved by only.06. The HUNT4 dataset is more standardized, contains recordings from mostly healthy volunteers and contains less variation than the CAMUS dataset. Thus, the increase in variation from the generative augmentations mostly benefits this dataset. However, table 3 shows that also the segmentation model trained on CAMUS and tested on HUNT4 shows small but statistically significant improvement. The proposed generative augmentations improve the variation in terms of acquisition, positioning and size of the LV in the image, but do not diversify in terms of shape of the heart itself. In practice, these two types of variation might be correlated, as more diversity in patients would naturally lead to more variation in scan sectors.

We consider the nnU-Net to be the current state-of-the-art for segmentation on CAMUS^7,25,26^ and used it as baseline for segmentation. Related works that use generative AI to aid in cardiac segmentation model training are designed in a setting where the goal is to generate a synthetic dataset that replaces the original dataset^11,13^. This is different from our approach, where the goal is to augment the existing dataset and improve segmentation performance. Nevertheless, table 3 also includes the results from Gilbert et al.^11^ and Stojanovski et al.^13^ for completion.

**Fig. 13:**
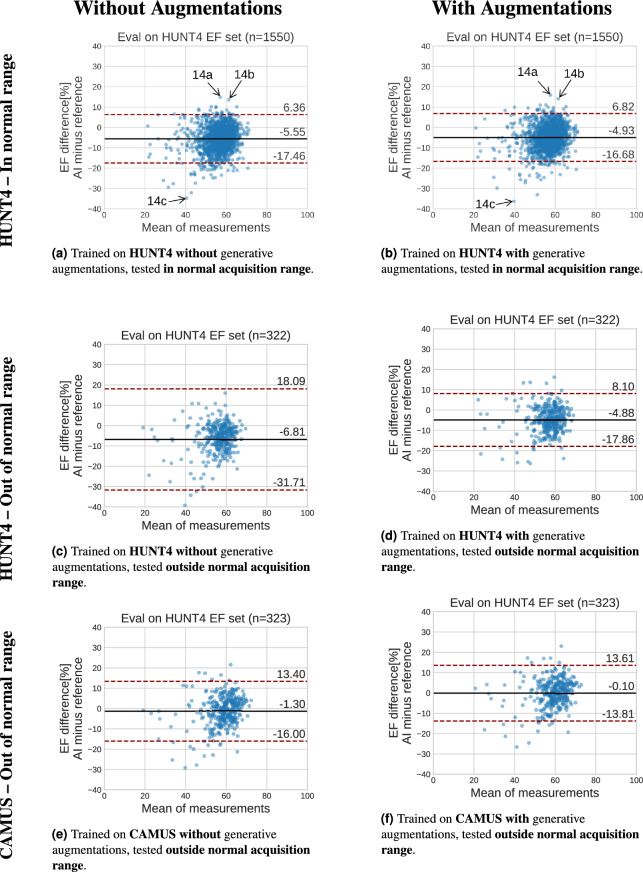
Bland–Altman plots comparing the manual reference with automatic EF measurements obtained via segmentation trained with and without generative augmentations. The exams outside the normal range are the exams where at least one frame used in the calculation is outside the normal range of HUNT4 (depth $>150$mm or sector angle $>70^\circ$). Figure 14 shows frames from the outliers indicated in the top two plots.

**Fig. 14:**
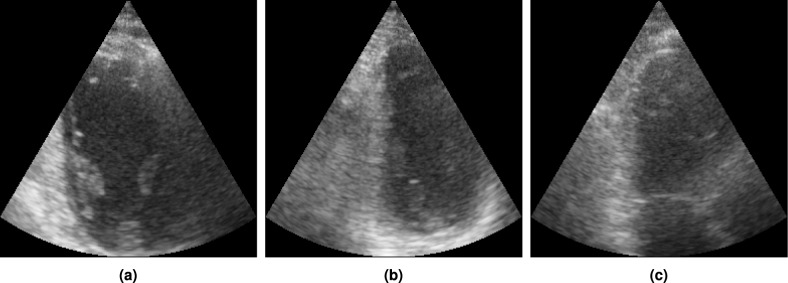
Examples of ED or ES frames from recordings in the HUNT4 development set that are outliers in the Bland–Altman plots 13a and 13b.

Figure 13 shows that the automatic EF measurements obtained with segmentation tend to underestimate the EF compared to the reference values. This consistent negative bias is not trivial to explain, since it could be caused by a range of factors. It can originate from limitations in the deep learning approaches themselves or potential inaccuracies in the reference data. Olaisen et al.^4^ gives a more in-depth discussion. The failing cases in the clinical evaluation were mainly cases where the image view was suboptimal or when the image quality was poor, as also reported in our previous work^26^. The plots in Fig. 13 show that the results on HUNT4 are similar in the acquisition normal range, indicating that the generative augmentations do not improve the EF measurement accuracy substantially on these data. Some of the outliers of the model without generative augmentations are also outliers for the model with generative augmentations. Figure 14 shows the relevant frames according to these outliers. They all have in common that the anterior wall is difficult to trace. The endocardial borders used to extract the volumes were not saved, so it is not possible to compare it directly to the one extracted by the segmentation model. Testing outside the acquisition normal range of depth> 150 mm and angle > 70, the generative augmentations clearly improve the results.

### Limitations and future potential

The main limitations of our work are: 1) exclusion of the LA in segmentation, and 2) long inference times for augmentations due to the iterative nature of the diffusion model. The LA is often excluded because it’s not fully visible in many images. Depth augmentation can generate missing atrial parts, but this invalidates the original mask, unless the full atrium is present and only unmasked regions are altered. As for inference time, the diffusion model is not optimized for speed, taking $\sim$1 minute per sample and $\sim$17 minutes for 32 samples on an Nvidia L40S GPU. Therefore, augmentations were precomputed before training rather than applied on-the-fly.

This study explores generative augmentations for cardiac image segmentation. While tailored to ultrasound, the approach is adaptable to other tasks and imaging modalities. This is especially useful when traditional augmentations can not realistically alter mask positions or image content. Adapting it to a new modality would require training a modality-specific diffusion model and implementing relevant transformations. For example, in MRI or CT, a tumour could be relocated within an organ, with the model regenerating realistic surroundings. Although trained on the CAMUS dataset, the unconditional generative model could also learn from unlabelled data and capture broader variability to augment smaller labelled datasets.

## Conclusion

The main contribution in this work is the introduction of generative augmentations for improving cardiac ultrasound segmentation. We show that diffusion models can generate highly realistic cardiac ultrasound images that can be used to improve segmentation model accuracy and generalizability through generative augmentations. Notably, the improvements in segmentation accuracy and EF estimation were achieved without altering the underlying machine learning architecture, demonstrating the effectiveness of the augmentation strategy alone.

The effectiveness of the generated samples was validated qualitatively by experts through visual inspection and quantitatively through improved Dice score, Hausdorff distance and EF estimation. The proposed generative augmentations are most useful for datasets with limited variation in terms of acquisition depth and width, and positioning of the LV in the image. This is relevant in the medical domain, as ultrasound datasets are often limited according to the acquisition protocol and personal preferences at a given clinic.

In conclusion, this study provides evidence that generative augmentation using diffusion models can be used for enhancing model robustness and generalizability for echocardiography segmentation.

## Supplementary Information

Supplementary Information.

## Data Availability

The CAMUS dataset is publicly available^7^. The HUNT4 dataset is an in-house dataset from St. Olavs hospital in Trondheim, Norway and is currently not publicly available. All participants signed informed consent for participation and the use of data in research^29^. The REB approved all parts of the study (REC ID 2018/2416). The datasets used and analyzed during the current study is not public available. Håvard Dalen (havard.dalen@ntnu.no) is the contact point for the HUNT4Echo data.
