# Supplementary material for: Generative augmentations for improved cardiac ultrasound segmentation using diffusion models
Source: Sci Rep. 2025 Oct 30;15:38013. doi: 10.1038/s41598-025-21938-y (PMC12575775; doi:10.1038/s41598-025-21938-y)
Supplement: Supplementary file 1 — Supplementary Information. [file 41598_2025_21938_MOESM1_ESM.pdf]

Supplementary material : Extensive evaluation of automatic EF

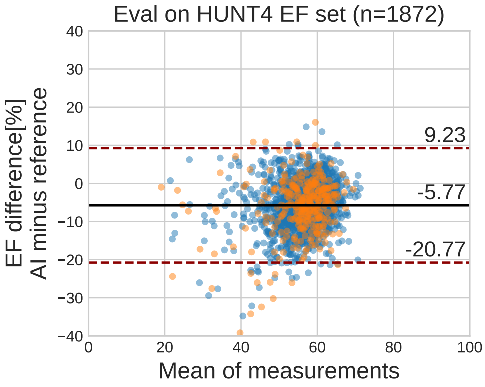

(a) Trained on **HUNT4 without** generative augmentations

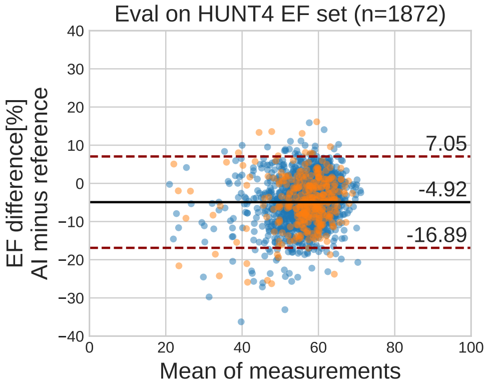

(b) Trained on **HUNT4 with** generative augmentations

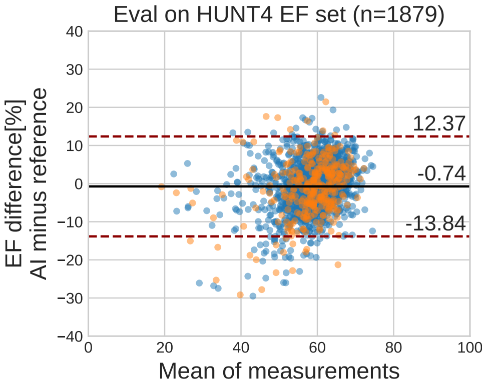

(c) Trained on **CAMUS without** generative augmentations

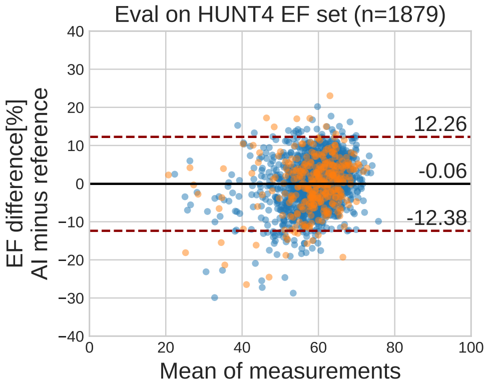

(d) Trained on **CAMUS with** generative augmentations

Figure 1. Evaluation of automatic EF on the **HUNT4 EF set** obtained via segmentation models trained with and without generative augmentations. The orange dots represent exams where at least one frame used in the calculation is outside the normal range for HUNT4 (depth $>$ 150mm or sector angle $> 70°$). The reference EF values are obtained using EchoPAC software (GE HealthCare).

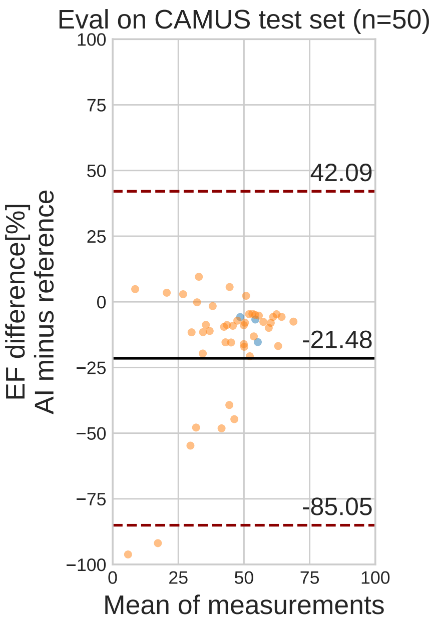

(a) Trained on **HUNT4 without** generative augmentations

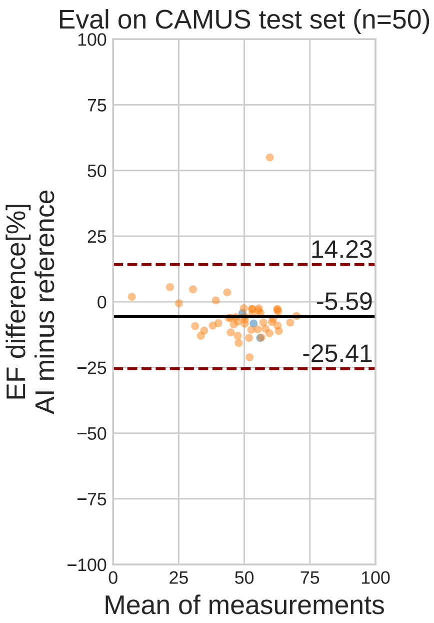

(b) Trained on **HUNT4 with** generative augmentations

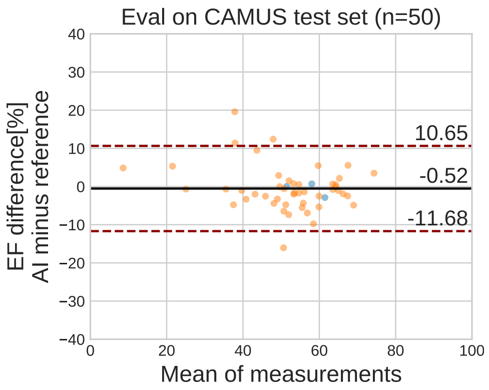

(c) Trained on **CAMUS without** generative augmentations

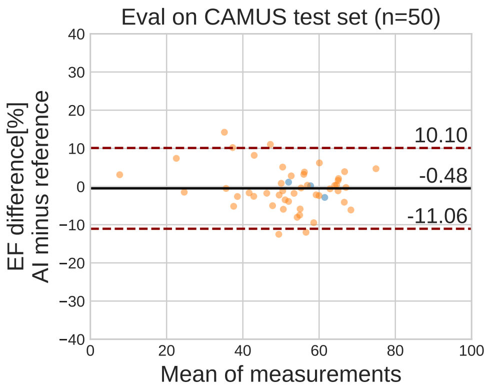

(d) Trained on **CAMUS with** generative augmentations

Figure 2. Evaluation of automatic EF on **CAMUS** obtained via segmentation models trained with and without generative augmentations. The orange dots represent exams where at least one frame used in the calculation is outside the normal range for HUNT4 (depth $>$ 150mm or sector angle $>$ 70°). The reference EF values are obtained using the automatic EF algorithm[1,2] with the reference segmentation masks.

Supplementary material: Technical details on neural network architectures and training setup

Table 1 summarizes the technical details of the U-Net used in the diffusion model. Table 2 summarizes the technical details of the nnU-Net used for segmentation.

Table 1. Key characteristics of the U-Net and its training setup in the diffusion model. The "number of channels" row refers to the number of channels at the first, bottom, and final convolution layers of the U-Net architecture. The "Residual blocks" row refers to the number of blocks per spatial resolution level. For more details, see Nichol and Dhariwal[3,4].

Number of parameters	44.1 million
Input size	256×256
Number of channels	$64 \downarrow 256 \uparrow 64$
Lowest resolution	8×8
Upsampling scheme	Nearest neighbour interpolation
Downsampling scheme	Average pooling
Normalization scheme	GroupNorm
Batch Size	64
Optimizer	Adam
Learning rate	1e-4
Learning rate scheduler	None
Activation	SiLU
Residual blocks	3
Training steps	500k
Self-attention	At resolutions 8 and 16, 4 heads
Diffusion steps	4000
Noise scheduler	Cosine[4]
Learn variance	Yes
Loss	Mean squared error, corresponding to the L_{simple} learning objective in[3].

Table 2. Key characteristics of the nnU-Net used for segmentation[5,6]. The "number of channels" row refers to the number of channels at the first, bottom, and final convolution layers of the U-Net architecture. The "Residual blocks" row refers to the number of blocks per spatial resolution level. The regular augmentations listed here are performed on top of the proposed generative augmentations. For more details, see Isensee et al.[6].

Number of parameters	33.4 million
Input size	256×256
Number of channels	$32 \downarrow 512 \uparrow 32$
Lowest resolution	4×4
Upsampling scheme	Transposed convolutions
Downsampling scheme	Strided convolutions
Normalization scheme	InstanceNorm
Batch Size	49
Optimizer	Adam
Initial learning rate	1e-2
Learning rate scheduler	Polynomial annealing
Loss function	Dice & cross-entropy
Inter-layer activation	Leaky ReLU
Final layer activation	Softmax
Residual blocks	2
Epochs	500
Deep supervision	At resolutions 128, 64, 32, and 16
Augmentations	Rotations, scaling, Gaussian noise, Gaussian blur, brightness, contrast, simulation of low resolution, gamma correction, and mirroring.

References

1. Smistad, E. *et al.* Real-time automatic ejection fraction and foreshortening detection using deep learning. *IEEE transactions on ultrasonics, ferroelectrics, frequency control* **67**, 2595–2604 (2020).
2. Van De Vyver, G. *et al.* Towards robust cardiac segmentation using graph convolutional networks. *IEEE Access* (2024).
3. Dhariwal, P. & Nichol, A. Diffusion models beat gans on image synthesis. *Adv. neural information processing systems* **34**, 8780–8794 (2021).
4. Nichol, A. Q. & Dhariwal, P. Improved denoising diffusion probabilistic models. In *International conference on machine learning*, 8162–8171 (PMLR, 2021).
5. Isensee, F., Jaeger, P. F., Kohl, S. A., Petersen, J. & Maier-Hein, K. H. nnu-net: a self-configuring method for deep learning-based biomedical image segmentation. *Nat. methods* **18**, 203–211 (2021).
6. Isensee, F., Jaeger, P. F., Kohl, S. A., Petersen, J. & Maier-Hein, K. H. nnunet. https://github.com/MIC-DKFZ/nnUNet (2023).
